# Antihypertensive drug treatment changes in the general population: the colaus study

**DOI:** 10.1186/2050-6511-15-20

**Published:** 2014-03-31

**Authors:** Vanessa Christe, Gérard Waeber, Peter Vollenweider, Pedro Marques-vidal

**Affiliations:** 1 Institute of Social and Preventive Medicine (IUMSP), Lausanne University Hospital, Bâtiment Biopôle 2, Route de la Corniche 10, 1010 Lausanne, Switzerland; 2 Department of Medicine, Internal Medicine, Lausanne University Hospital (CHUV) and Faculty of biology and medicine, Lausanne, Switzerland

**Keywords:** Antihypertensive drug therapy, Prospective study, Switzerland, Switching, Persistence, Blood pressure, Combination, Discontinuation

## Abstract

**Background:**

Changes in antihypertensive drug treatment are paramount in the adequate management of patients with hypertension, still, there is little information regarding changes in antihypertensive drug treatment in Switzerland. Our aim was to assess those changes and associated factors in a population-based, prospective study.

**Methods:**

Data from the population-based, CoLaus study, conducted among subjects initially aged 35–75 years and living in Lausanne, Switzerland. 772 hypertensive subjects (371 women) were followed for a median of 5.4 years. Data Subjects were defined as continuers (no change), switchers (one antihypertensive class replaced by another), combiners (one antihypertensive class added) and discontinuers (stopped treatment). The distribution and the factors associated with changes in antihypertensive drug treatment were assessed.

**Results:**

During the study period, the prescription of diuretics decreased and of ARBs increased: at baseline, diuretics were taken by 46.9% of patients; angiotensin receptor blockers (ARB) by 44.7%, angiotensin converting enzyme inhibitors (ACEI) by 28.8%, beta-blockers (BB) by 28.0%, calcium channel blockers (CCB) by 18.9% and other antihypertensive drugs by 0.3%. At follow-up (approximately 5 years later), their corresponding percentages were 42.8%, 51.7%, 25.5%, 33.0% 20.7% and 1.0%. Among all participants, 54.4% (95% confidence interval: 50.8-58.0) were continuers, 26.9% (23.8-30.2) combiners, 12.7% (10.4-15.3) switchers and 6.0% (4.4-7.9) discontinuers. Combiners had higher systolic blood pressure values at baseline than the other groups (p < 0.05). Almost one third (30.6%) of switchers and 29.3% of combiners improved their blood pressure status at follow-up, versus 18.8% of continuers and 8.7% of discontinuers (p < 0.001). Conversely, almost one third (28.3%) of discontinuers became hypertensive (systolic ≥140 mm Hg or diastolic ≥90 mm Hg), vs. 22.1% of continuers, 16.3% of switchers and 11.5% of combiners (p < 0.001). Multivariate analysis showed baseline uncontrolled hypertension, ARBs, drug regimen (monotherapy/polytherapy) and overweight/obesity to be associated with changes in antihypertensive therapy.

**Conclusion:**

In Switzerland, ARBs have replaced diuretics as the most commonly prescribed antihypertensive drug. Uncontrolled hypertension, ARBs, drug regimen (monotherapy or polytherapy) and overweight/obesity are associated with changes in antihypertensive treatment.

## Background

Hypertension is an important manageable risk factor of Cardiovascular Diseases (CVD), a major cause of morbidity and mortality worldwide [1], and its prevalence has been estimated at 36% in Switzerland [2]. Hypertension has considerable humanistic and economic consequence [3] and an effective and appropriate treatment must be provided to achieve blood pressure (BP) levels < 140/90 mmHg [4].

In many cases, a lifetime antihypertensive drug treatment is recommended [3] and combination therapy is often necessary to achieve BP control [5]. However, poor adherence to antihypertensive drug treatment has repeatedly been showed: in a Canadian study, 55% of participants on diuretics discontinued treatment after 1 year [6], and a similar discontinuation rate (53%) was found in Italy [7]. The absence of clinical symptoms of hypertension identifiable by the patient along with a low tolerability of certain antihypertensive drugs are the most common explanations why patients stop their treatment or take their medication at inappropriate intervals or wrong doses [3].

In a previous study [2], we assessed the prevalence and management of hypertension in Switzerland. Still, there is little if no information regarding changes in or discontinuation of antihypertensive drug treatment in this country. The aim of this study was thus to assess the therapeutic changes in hypertensive participants treated over a period of approximately five years using data from a population-based, prospective study and to identify the factors associated with those changes.

## Methods

### The CoLaus study

The sampling procedure of the Cohorte Lausannoise (CoLaus) study has been described previously [8]. The CoLaus study has been accepted by the Ethics Committee of the Canton Vaud and aims at assessing the genetic determinants of cardiovascular disease in the Caucasian population of Lausanne. The non-genetic part of the CoLaus study included all participants, irrespective of their ethnicity. Hence, only Caucasians were included in the main study to avoid population stratification and to increase genetic homogeneity for association studies. Still, non Caucasian subjects were also examined (but not included in the main study). The following inclusion criteria were applied: (i) written informed consent; (ii) age 35–75 years; (iii) willingness to take part in the examination and to have a blood sample drawn. Recruitment began in June 2003 and ended in May 2006. Quickly, the complete list of the Lausanne inhabitants aged 35–75 years (n = 56,694) was provided by the population registry of the city and a simple, nonstratified random sample of 35% was drawn. An invitation letter with a quick description of the study was sent to all randomized participants. Interested individuals were contacted telephonically within 14 days by one of the staff members who provided more information about the study and arranged for an appointment. Participation rate was 41% and 6,733 participants (3,544 women and 3,189 men) were recruited. In this study, all participants, irrespective of their ethnicity, were included.

### Baseline risk factor assessment

All participants attended the outpatient clinic of the University Hospital of Lausanne in the morning after an overnight fast (minimum fasting time 8 hours). Data were collected by trained field interviewers in a single visit lasting about 60 min.

Participants received a questionnaire to record information about their status and lifestyle factors. Educational level was stratified into basic, apprenticeship, secondary school and university. Smoking status was classified as never, current or former smoker. Physical activity was defined as the practice of leisure time physical activity at least twice per week. During a face-to-face meeting, family history (mother and father) of myocardial infarction, stroke, hypertension, dyslipidemia or diabetes was collected. No data was available for other first degree relatives such as parents and siblings . Participants were also asked if they had previously experienced myocardial infarction, stroke or any other type of cardiovascular disease such as angina or peripheral artery disease. Personal history of and current treatment for hypercholesterolemia or diabetes were also determined. Information on the use of prescription and over the counter drugs was collected, together with their main indications. Collection was done by asking the participant to bring the drugs to the visit and the number of non-antihypertensive drugs prescribed was assessed.

Body weight and height were measured in light indoor clothes with shoes off. Body weight was measured in kilograms to the nearest 100 g using a Seca® scale, which was calibrated regularly. Height was measured to the nearest 5 mm using a Seca® height gauge. Body Mass Index (BMI) was calculated as weight (kg) divided by the square of the height (m). Overweight was defined by a BMI ≥25 kg/m^2^ and <30 kg/m^2^, and obesity by a BMI ≥30 kg/m^2^. A similar procedure was performed at follow-up. Waist was measured with a non-stretchable tape over the unclothed abdomen at the narrowest point between the lowest rib and the iliac crest. Two measures were made and the mean (expressed in centimeters) used for analyses. Abdominal obesity was considered for a waist ≥ 102 cm for men and ≥ 88 cm for women [9].

After a median follow-up time of 5.4 years (interquartile range: 5.3–5.6 years), participants were invited to attend a second examination, which included the same assessments as for baseline.

### Antihypertensive drug treatment and blood pressure status

The names of all antihypertensive drugs were collected and coded using the Anatomical Therapeutic Chemical (ATC) classification system [10]. In both baseline and follow-up, antihypertensive drugs were classified into six different categories: 1) Diuretics (isolated or associated with other drugs); 2) Calcium channel blockers (CCBs); 3) Beta-blockers (BBs); 4) Angiotensin-converting enzyme inhibitors (ACEIs); 5) Angiotensin receptor blockers (ARB) and 6) Other (reserpine). Combinations were split into the drug classes they contained; for example ATC code C08GA01, corresponding to nifedipine and diuretics, was split into “diuretics associated with other drugs” and “calcium channel blockers”. As a single medicine can be a combination of up to three antihypertensive drug classes, two further classifications were used according to the number of antihypertensive drug classes or of antihypertensive pills: monotherapy (i.e. taking a single drug class)/combination therapy and single medicated (i.e. taking a single pill, which can eventually be a combination of drugs)/polymedicated.

According to the evolution of their antihypertensive treatment, participants were assigned into 4 different groups determined by drugs brought to visit at baseline and approximately 5 years later as suggested in a previous study [7]: 1) *Continuers*: participants continuing the initial treatment (including combinations) without changes; 2) S*witchers*: treatment from one class to another class of antihypertensive therapy (for example a CCB for an ARB); 3) *Combiners*: participants treated with an additional type of antihypertensive class but continuing the initial medication (for example adding a diuretic to an ARB) and 4) *Discontinuers*: participants stopping the therapy without having another antihypertensive drug prescription added.

On baseline and follow-up, BP was measured on the left arm, with an appropriately sized cuff. The reading was taken following at least 10 minute rest in the seated position, using an Omron® HEM-907 automated oscillometric sphygmomanometer. Three readings were taken and the average of the last two was used to compute systolic (SBP) and diastolic (DBP) blood pressure. A participant was considered as adequately controlled if her/his SBP was <140 mm Hg and her/his DBP was <90 mm Hg in the absence of diabetes, and if her/his SBP was <130 mm Hg and her/his DBP was <80 mm Hg in the presence of diabetes [11].

### Statistical analysis

Statistical analyses were completed using Stata v.12.0 (Stata Corp, College Station, TX, USA). Results were expressed as number of participants (percentage) or as mean ± standard deviation. Between-group comparisons were performed using Chi-square for qualitative variables or Student’s t-test or one-way analysis of variance (ANOVA) for quantitative variables. Post-hoc analyses after ANOVA were conducted using the Scheffe method. Multivariate analysis was conducted using Multinomial (polytomous) logistic regression and the results were expressed as relative risk ratio and (95% confidence interval). Statistical significance was considered for p < 0.05.

## Results

### Sample’s characteristics

Among the 6,733 participants initially assessed, 4,973 (73.9%) had follow-up data at the present time, of which 772 (15.5%, 371 women) were treated for hypertension at baseline. Their clinical characteristics at baseline are summarized in Additional file 1: Table S1.

### Distribution of antihypertensive drug classes

Distribution of antihypertensive drug classes at baseline and follow-up are summarized in Table 1. At baseline, the main antihypertensive classes were diuretics (mainly in association with other antihypertensive drugs) and ARBs, followed by ACE inhibitors and BBs. At follow-up, the percentage of participants on ARBs and BBs increased while the percentage of participants on diuretics and ACE inhibitors decreased. At baseline, almost half of the patients were treated with a single antihypertensive class, and less than one sixth with 3 or more antihypertensive classes. At follow-up, the percentage of participants treated with a single antihypertensive class decreased, while the percentage of patients treated with three or more antihypertensive classes increased to one fifth (Table 1).

**Table 1:** Antihypertensive drug treatment at baseline and follow-up, CoLaus study

	**Baseline**	**Follow-up**
Diuretics (%)	392 (46.9)	330 (42.8)
As main treatment (%)	93 (12.1)	108 (14.0)
Associated with other drugs (%)	293 (38.0)	240 (31.1)
Angiotensin receptor blockers (%)	345 (44.7)	399 (51.7)
Angiotensin converting enzyme inhibitors (%)	222 (28.8)	197 (25.5)
Beta-blockers (%)	216 (28.0)	255 (33.0)
Calcium channel blockers (%)	146 (18.9)	160 (20.7)
Other (%)	2 (0.3)	8 (1.0)
Number of antihypertensive classes (%)		
0	-	46 (6.0)
1	368 (47.7)	293 (38.0)
2	296 (38.3)	270 (35.0)
3+	108 (14.0)	163 (21.0)

Results are expressed as number of participants and (percentage). The total number of participants (772) was used as denominator to calculate percentages.

### Changes in antihypertensive drug treatment

Among all (mono or combination therapy) participants, 54.4% (95% confidence interval: 50.8-58.0) were continuers, 26.9% (23.8-30.2) combiners, 12.7% (10.4-15.3) switchers and 6.0% (4.4-7.9) discontinuers (Table 2). Among participants on monotherapy, the results were 42.1% (37.0-47.3), 35.9% (31.0-41.0), 13.3% (10.0-17.2) and 8.7% (6.0-12.1).

**Table 2:** Baseline individual factors associated with antihypertensive drug changes, CoLaus study

	**Continuers**	**Combiners**	**Switchers**	**Discontinuers**	**p-value**
**N**	**420 (54.4)**	**208 (26.9)**	**98 (12.7)**	**46 (6.0)**	
Women (%)	213 (50.7)	93 (44.7)	50 (51.0)	15 (32.6)	0.08
Age (years)	60.2 ± 9.0	61.2 ± 8.9	59.2 ± 10.1	57.7 ± 8.2	0.06
Educational status (%)					
Basic	94 (22.4)	46 (22.1)	22 (22.5)	10 (21.7)	
Apprenticeship	186 (44.3)	90 (43.3)	37 (37.8)	18 (39.1)	0.90
High school/college	91 (21.7)	47 (22.6)	26 (26.5)	9 (19.6)	
University	49 (11.7)	25 (12.0)	13 (13.3)	9 (19.6)	
Smoking status (%)					
Never	169 (40.2)	75 (36.1)	43 (43.9)	19 (41.3)	
Former	160 (38.1)	94 (45.2)	35 (35.7)	18 (39.1)	0.67
Current	91 (21.7)	39 (18.8)	20 (20.4)	9 (19.6)	
Physically active (%)	205 (48.8)	100 (48.1)	56 (57.1)	26 (56.5)	0.34
BMI (kg/m^2^)	28.4 ± 4.9	29 ± 4.4	27.9 ± 4.9	27.7 ± 4.2	0.15
BMI categories (%)					
Normal	105 (25.0)	36 (17.3)	29 (29.6)	12 (26.1)	
Overweight	181 (43.1)	92 (44.2)	41 (41.8)	26 (56.5)	<0.05
Obese	134 (31.9)	80 (38.5)	28 (28.6)	8 (17.4)	
Waist (cm)	97 ± 14	99 ± 14	95 ± 13	96 ± 12	0.13
Abdominal obesity (%)	219 (52.1)	121 (58.2)	48 (49.0)	16 (34.8)	<0.05
Alcohol drinker (%)	301 (71.7)	160 (76.9)	74 (75.5)	31 (67.4)	0.39
Personal history of (%)					
Myocardial infarction	27 (6.4)	16 (7.7)	2 (2.0)	1 (2.2)	NA
Stroke	11 (2.6)	8 (3.9)	5 (5.1)	1 (2.2)	NA
CVD	52 (12.4)	35 (16.8)	11 (11.2)	6 (13.0)	0.41
Dyslipidemia	182 (43.3)	105 (50.5)	39 (39.8)	19 (41.3)	0.23
Diabetes	64 (15.2)	31 (14.9)	10 (10.2)	4 (8.7)	0.41
Family history of (%)					
Myocardial infarction	110 (26.2)	44 (21.2)	26 (26.5)	14 (30.4)	0.43
Stroke	88 (21.0)	35 (16.8)	23 (23.5)	10 (21.7)	0.51
Hypertension	200 (47.6)	106 (51.0)	49 (50.0)	27 (58.7)	0.51
Dyslipidemia	72 (17.1)	41 (19.7)	19 (19.4)	12 (26.1)	0.48
Diabetes	85 (20.2)	44 (21.2)	20 (20.4)	12 (26.1)	0.83
Number of other drugs ^§§^	2.9 ± 2.4	3.1 ± 2.5	3.0 ± 2.2	2.7 ± 2.7	0.80

Results are expressed as mean ± standard deviation or as number of participants and (percentage). ^§^defined as the practice of leisure time physical activity at least twice per week; ^§§^non-antihypertensive drugs prescribed. BMI, body mass index;. Statistical analysis comparing all groups by Chi-square or one-way analysis of variance: NA, not assessable.

**Figure 1:**
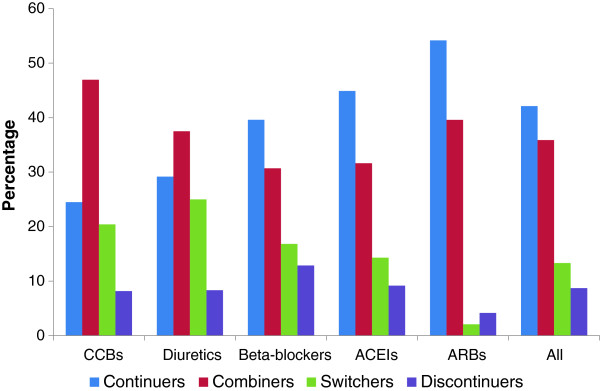
**Distribution of continuers, combiners, switchers and discontinuers, according to the pharmacological category of the antihypertensive drug, CoLaus study.** ACEIs, angiotensin converting enzyme inhibitors; ARBs, angiotensin receptor blockers; BBs, beta-blockers; CCBs, calcium channel blockers.

The distribution of continuers, combiners, switchers and discontinuers according to the pharmacological class of the antihypertensive drug is summarized in Figure 1 for participants on monotherapy only. The need for an additional antihypertensive class (combination) were higher in participants treated with CCBs (46.9%), ARBs (39.6%) and diuretics (37.5%). Participants treated with ARBs, ACEIs and BBs had a better continuation’s rate, with a percentage of 54.2%, 44.9% and 33.6% respectively. Finally, treatment switching was more common among participant using diuretics (25.0%), CCBs (20.4%) and BBs (16.8%).

### Factors associated with changes in antihypertensive drug treatment

The associations between changes in antihypertensive drug treatment and several personal, family and clinical characteristics at baseline are summarized in Tables 2 and 3. Prevalence of overweight/obesity and abdominal obesity differed between groups, but no differences were found regarding BMI or waist levels. Conversely, no differences were found between groups for personal and family history, number of other prescribed drugs and other clinical and biological variables (Table 2). SBP and DBP differed significantly between groups: combiners presented higher SBP values at baseline than the other groups (Scheffe’s p < 0.05), while for DBP only the difference between combiners and continuers was statistically significant. Combiners and discontinuers took diuretics less frequently, while switchers took ARBs less frequently. Combiners and discontinuers also had the highest prevalence of a one pill, single drug treatment (Table 3).

**Table 3:** Baseline blood pressure factors associated with antihypertensive drug changes, CoLaus study

	**Continuers**	**Combiners**	**Switchers**	**Discontinuers**	**p-value**
**N**	**420 (54.4)**	**208 (26.9)**	**98 (12.7)**	**46 (6.0)**	
BP status baseline					
SBP (mm Hg)	137 ± 17	146 ± 19	138 ± 16	137 ± 15	<0.001
DBP (mm Hg)	83 ± 11	86 ± 12	83 ± 11	83 ± 9	<0.05
Hypertension^§^	220 (52.4)	138 (66.4)	54 (55.1)	20 (43.5)	<0.001
Antihypertensive drug					
Diuretics (%)	236 (56.2)	64 (30.8)	47 (48.0)	15 (32.6)	<0.001
Beta-blockers (%)	120 (28.6)	50 (24.0)	30 (30.6)	16 (34.8)	0.38
CCB (%)	76 (18.1)	42 (20.2)	23 (23.5)	5 (10.9)	0.30
ACE inhibitors (%)	119 (28.3)	58 (27.9)	32 (32.7)	13 (28.3)	0.84
ARBs (%)	224 (53.3)	82 (39.4)	26 (26.5)	13 (28.3)	<0.001
Treatment regimen (%)					
One pill, single drug	155 (36.9)	132 (63.5)	49 (50.0)	32 (69.6)	
One pill, combination	119 (28.3)	34 (16.4)	26 (26.5)	7 (15.2)	<0.001
Several pills	146 (34.8)	42 (20.2)	23 (23.5)	7 (15.2)	

Results are expressed as mean ± standard deviation or as number of participants and (percentage). ^§^defined as a systolic blood pressure ≥140 mm Hg or diastolic blood pressure ≥90 mm Hg if absence of diabetes, or as a systolic blood pressure ≥130 mm Hg or diastolic blood pressure ≥80 mm Hg if presence of diabetes. CCB, calcium channel blockers; ACE, angiotensin converting enzyme; ARB, angiotensin receptor blockers. Statistical analysis comparing all groups by Chi-square or one-way analysis of variance.

The results of the multivariate analysis of the factors associated with changes in antihypertensive drug treatment relative to continuers group are summarized in Table 4. Compared to continuers, discontinuers were less likely to be women, to take ARBs and more likely to be on a single pill, single drug regimen. Combiners were more likely to present with uncontrolled blood pressure at baseline, to be on a single pill, single drug regimen and to be overweight or obese. Switchers were less likely to take ARBs (Table 4). No association was found between antihypertensive drug changes and being on a single pill, combination regimen (Table 4). Similarly, in an extended model, no association was found between changes in antihypertensive drug treatment and marital status, educational level, smoking status, being physically active, personal history of CVD, age or number of other non-antihypertensive drugs prescribed (not shown).

**Table 4:** Multivariate analysis of the baseline factors associated with changes in antihypertensive drug treatment, CoLaus study

	**Combiners**	**Switchers**	**Discontinuers**
Women vs. men	0.79 (0.55 - 1.12)	0.94 (0.60 - 1.48)	0.42 (0.21 - 0.81)
ARB (yes vs. no)	0.77 (0.53 - 1.13)	0.31 (0.18 - 0.52)	0.48 (0.23 - 0.98)
Hypertension (yes vs. no)	1.69 (1.18 - 2.42)	1.09 (0.69 - 1.72)	0.66 (0.35 - 1.23)
Treatment regimen			
Several pills	1 (ref)	1 (ref)	1 (ref)
One pill, combination	1.06 (0.63 - 1.79)	1.78 (0.94 - 3.38)	1.48 (0.49 - 4.47)
One pill, single drug	3.21 (2.06 - 5.00)	1.55 (0.88 - 2.74)	4.06 (1.68 - 9.83)
BMI status			
Normal	1 (ref)	1 (ref)	1 (ref)
Overweight	1.83 (1.13 - 2.95)	0.95 (0.55 - 1.66)	1.52 (0.71 - 3.24)
Obese	2.35 (1.42 - 3.88)	0.96 (0.53 - 1.76)	0.76 (0.29 - 1.98)

ARB, angiotensin receptor blocker; BMI, body mass index. Statistical analysis by multinomial (polytomous) logistic regression, using continuers as the reference group. Hypertension was defined as a systolic blood pressure ≥140 mm Hg or a diastolic blood pressure ≥90 mm Hg. Results are expressed as relative risk ratio and (95% confidence interval).

### Impact of changes in antihypertensive drug treatment on blood pressure status

Compared to continuers, combiners and switchers improved their blood pressure status at follow-up (p < 0.001, Table 5). Among continuers, 18.6% improved their blood pressure while 20.7% worsened (net result: 2.1% worsening); the corresponding values for combiners were 28.9% and 13.0% (net result: 15.9% improvement) and for switchers 30.6% and 14.3% (net result: 16.3% improvement). Finally, 8.7% of discontinuers improved their blood pressure while 26.1% worsened (net result: 17.6% worsening, Table 5).

**Table 5:** Evolution of blood pressure status according to changes in antihypertensive drug treatment, CoLaus study

	**Continuers**	**Combiners**	**Switchers**	**Discontinuers**
**N**	**420**	**208**	**98**	**46**
C to HT	87 (20.7)	27 (13.0)	14 (14.3)	12 (26.1)
HT to HT	142 (33.8)	78 (37.5)	24 (24.5)	16 (34.8)
C to C	113 (26.9)	43 (20.7)	30 (30.6)	14 (30.4)
HT to C	78 (18.6)	60 (28.9)	30 (30.6)	4 (8.7)

Results are expressed as number of participants and (percentage). C, controlled blood pressure (systolic <140 mm Hg and diastolic <90 mm Hg); HT, hypertension (systolic ≥140 mm Hg or diastolic ≥90 mm Hg). Statistical analysis by Chi-square comparing all BP evolutions between groups: p < 0.001.

Continuers with uncontrolled BP at follow-up were more frequently men (54.1% vs. 44.6, p = 0.05), were more frequently uncontrolled (55.5% vs. 37.4%, p < 0.001) and on diuretics at baseline (15.8% vs. 8.5%, p < 0.05), while no differences were found for the other variables (not shown).

## Discussion

### Distribution of antihypertensive drug classes

The most commonly prescribed antihypertensive drug classes were ARBs and diuretics, a finding in agreement with another Swiss study [4]. The prescription rate for ARBs at follow-up was considerably higher than reported in other countries (51.7% vs. 18-36%) [12] but in accordance with the guidelines of the Swiss Society of Hypertension [13] and others [12]. Indeed, the Swiss guidelines recommend that ACEIs, ARBs, CCBs and diuretics be first line antihypertensives, BBs being considered second line drugs. A possible reason could be the choice of practitioners to prescribe an antihypertensive class with the lowest side effects but just as effective as the others [4]; further, the fact that antihypertensive medication is reimbursed by the Swiss health system might also induce practitioners to choose better tolerated, albeit more expensive antihypertensive drugs. Conversely, the low rate of ACE inhibitor prescriptions was not strictly in keeping with the guideline recommendations at baseline [11,12], which consider ACEIs as first-line antihypertensive drugs. This can be explained by the fact that the guidelines recommend not to combine ACE inhibitors with ARBs for the treatment of hypertension [12]. As ARBs are the most commonly prescribed drug in our study, this might have led to a decrease in the number of ACE inhibitor prescriptions.

The recent 2013 ESH/ESC guidelines on management of arterial hypertension indicate that diuretics, beta-blockers, calcium antagonists, ACEI and ARBs are all suitable for the initiation and maintenance of antihypertensive treatment [13]. Still, after an approximate follow-up of five years, our results show that diuretics were more frequently replaced and ARBs were more frequently prescribed. Thus, our results suggest that practitioners on everyday’s practice tend to switch from diuretics to ARB treatment.

### Changes in antihypertensive drug treatment

Almost four out of ten participants (39.6%) changed their antihypertensive drug regimen during an approximately 5.4 year follow-up (26.9% combination and 12.7% switching), a value higher than reported previously (18%) [4]. Conversely, the rate of discontinuers was very low compared to other studies (Additional file 1: Table S2). Possible reasons include more motivated, health-conscious participants, the prescription of better tolerated antihypertensive drugs, and the reimbursement of any type of antihypertensive drug by the Swiss health insurances. Further, it has been shown that a high proportion of patients discontinuing treatment are returning on therapy within 1 year [6]. Hence, it is possible that the high discontinuation rates reported in other studies [7,14] might be overestimated due to a short follow-up time. Overall, our results suggest that, contrary to other countries, antihypertensive drug treatment maintenance is very high in Switzerland when assessed over a period of years.

Discontinuation of diuretics was higher than all other antihypertensive drugs. This is likely to be associated with the well described side effects such as hypotension and/or sodium or potassium abnormalities and/or metabolic disturbance. All side effects known to be associated with ARBs, ACEI, CCB or BB were not specifically recorded for this large population-based study but the discontinuation rate is strictly in agreement with other studies [15,16] (Additional file 1: Table S3; for a review, see [3]) and in accordance with adverse effects well established in several studies [3,7-19].

### Factors associated with changes in antihypertensive drug treatment

Presence of uncontrolled hypertension was positively associated with antihypertensive drug combination, a finding also reported elsewhere [7]. These findings are in agreement with the guidelines of the Swiss Society of Hypertension [13] and others [20] which indicate that combination therapy should be prescribed if monotherapy fails to control blood pressure levels.

Being treated by ARBs was negatively associated with switching or discontinuing antihypertensive drug treatment, a finding in agreement with the literature [7]. The most likely explanation is the lower rate of adverse effects of ARBs relative to the other antihypertensive drugs [19].

Being on a one pill, single drug regimen was positively associated with combining or discontinuing treatment. Indeed, the single drug regimen might favor discontinuation because of fewer co-morbidities and the fact that most patients are symptom-free and might experience more side effects from the treatment than the disease itself [7].

Women had a lower risk of discontinuing antihypertensive drug treatment, a finding in agreement with some studies [21,22] but not with others [23]. Contrary to previous studies [14,24], no association was found between antihypertensive drug changes and smoking, physical activity, marital status, educational level, personal history of cardiovascular diseases. These findings suggest that changes in anti-hypertensive drug treatment are mainly due to factors related to blood pressure and/or to possible side effects of antihypertensive drug treatment rather than to the socio-economic status of the patients.

### Impact of changes in antihypertensive drug treatment on blood pressure status

Unlike larger studies [7,14,25], our study was able to assess the impact of antihypertensive drug treatment on blood pressure control. Overall, our results confirm that adjusting the antihypertensive drug regimen leads to favorable changes in blood pressure status. Conversely, discontinuing treatment leads to a deleterious increase in blood pressure levels, which could partly explain the greater incidence of CVD events among discontinuers [25].

Continuers with uncontrolled blood pressure at follow-up were more frequently men, with uncontrolled blood pressure and on diuretics at baseline. These findings suggest that diuretics might be less effective in controlling blood pressure than the other antihypertensive drugs, or that their side effects might lead to a lower compliance and thus worse BP control. Indeed, diuretics have been shown to have the lowest persistence rate of all antihypertensive drugs (Additional file 1: Table S3). They also indicate that practitioners should be more aggressive towards uncontrolled hypertension, as continuing the same treatment will not improve blood pressure control.

### Strengths and limitations

The main strength of this study is that it is population-based and used a representative sample of subjects with hypertension. Hence, the conclusions are applicable to the general population and to daily clinical practice compared to those from randomized controlled trials. This study also allowed the analysis of a considerable number of factors associated with antihypertensive drug changes. Further, several studies that assessed changes in antihypertensive drug treatment used only two [1,26,27] or three categories such as “continuers”, “switchers” and “discontinuers” [14]. In this study, we opted for a four-category classification as suggested by Mazzaglia and colleagues [7] because it reflected more accurately the behavior of a practitioner when managing a patient with hypertension. Indeed, our results suggest that the factors associated with combining antihypertensive drugs are different from those associated with maintenance of the antihypertensive drug regimen.

This study has also some limitations. Generalization might be limited by the modest participation rate (41%), but this rate is comparable to other epidemiological studies as reported by Wolff and colleagues [28]. It is also possible that the CoLaus participants are more health-conscious than the general population, thus biasing the observed prevalence of discontinuers and data on past medical history and clinical features. Unlike other studies [29], no record of adverse effects was available; hence, the impact of adverse effects of antihypertensive drugs could not be assessed. Further, it was not possible to objectively assess adherence to treatment. Compared to other studies [14,25], our sample size was rather small but blood pressure data was available while in the other studies it was not. Hence, the effect of antihypertensive drug changes could be objectively assessed, while the other studies lacked such information or relied on administrative data only. Participants might present with outdated prescription boxes or may forget to bring a box with them. This may have led to misclassification of the patient’s baseline drug therapy status. No information was available regarding dosage of antihypertensive drugs at baseline; thus, no analysis of possible dosage escalation could be performed. Similarly, for logistic and economic reasons no yearly follow-up of the cohort could be performed, so it is possible that therapy adjustments and interventions may have been missed. Although incidence of CVD events was available, it was not possible to establish whether the changes in antihypertensive drug treatment occurred before or after the occurrence of the CVD event. The next follow-up of the cohort will start in April 2014 and will allow evaluating the impact of antihypertensive drug treatment changes in preventing CVD events Finally, we do not know the precise reason(s) for discontinuation, namely if it was a patient or practitioner decision.

## Conclusion

In Switzerland, ARBs have replaced diuretics as the most commonly prescribed antihypertensive drug. The percentage of patients with hypertension who discontinue their treatment is considerably lower than in other countries. Uncontrolled hypertension, ARBs, drug regimen (monotherapy or polytherapy) and overweight/obesity are associated with changes in antihypertensive drug treatment.

## Competing interests

PV and GW received a research grant from GlaxoSmithKline to conduct the CoLaus baseline Study. The other authors (VC and PMV) indicate no conflict of interest.

## Authors’ contributions

VC completed part of the statistical analyses and wrote most of the article; PMV collected data, completed part of the statistical analysis and wrote part of the article; PV and GW revised the article for important intellectual content. PMV had full access to the data and is the guarantor of the study. All authors read and approved the final manuscript.

## Pre-publication history

The pre-publication history for this paper can be accessed here:

http://www.biomedcentral.com/2050-6511/15/20/prepub

## Supplementary Material

Additional file 1: Table S1 Baseline characteristics of CoLaus participants treated for hypertension (n=772). **Table S2.** Comparison of the proportion of continuers, combiners, switchers, and discontinuers in several studies. **Table S3.** Persistence with initial treatment in different studies. Click here for file
