# Supplementary material for: Antihypertensive drug treatment changes in the general population: the colaus study
Source: BMC Pharmacol Toxicol. 2014 Mar 31;15:20. doi: 10.1186/2050-6511-15-20 (PMC4021828; doi:10.1186/2050-6511-15-20)
Supplement: Additional file 1: Table S1 — Baseline characteristics of CoLaus participants treated for hypertension (n=772). Table S2. Comparison of the proportion of continuers, combiners, switchers, and discontinuers in several studies. Table S3. Persistence with initial treatment in different studies. [file 2050-6511-15-20-S1.doc]

**Supplementary files**

**Supplementary table 1:** baseline characteristics of CoLaus participants treated for hypertension (n=772).

| Men (%) | 401 (51.9) |
| --- | --- |
| Age (years) | 60.2 ± 9.1 |
| Educational status (%) |  |
| Basic | 172 (22.3) |
| Apprenticeship | 331 (42.9) |
| High school/ college | 173 (22.4) |
| University | 96 (12.4) |
| Smoking status (%) |  |
| Never | 306 (39.6) |
| Former | 307 (39.8) |
| Current | 159 (20.6) |
| Physically active (%) | 387 (50.1) |
| BMI (kg/m^2^) | 28.4 ± 4.7 |
| BMI categories (%) |  |
| Normal | 182 (23.6) |
| Overweight | 340 (44.0) |
| Obese | 250 (32.4) |
| Waist (cm) | 97.1 ± 13.6 |
| Abdominal obesity (%) | 404 (52.3) |
| Alcohol drinker (%) | 566 (73.3) |
| Personal history of (%) |  |
| Myocardial infarction | 46 (6.0) |
| Stroke | 25 (3.2) |
| Cardiovascular disease | 104 (13.5) |
| Dyslipidemia | 345 (44.7) |
| Diabetes | 109 (14.1) |
| Family history of (%) |  |
| Myocardial infarction | 194 (25.1) |
| Stroke | 156 (20.2) |
| Hypertension | 382 (49.5) |
| Dyslipidemia | 144 (18.6) |
| Diabetes | 161 (20.8) |
| Blood pressure status |  |
| SBP (mm Hg) | 140 ± 18 |
| DBP (mm Hg) | 84 ± 11 |
| SBP≥140 or DBP≥90 (%) | 397 (51.4) |
| Antihypertensive drug |  |
| Diuretics (%) | 362 (46.9) |
| Beta-blockers (%) | 216 (28.0) |
| CCB (%) | 146 (18.9) |
| ACE inhibitors (%) | 222 (28.8) |
| ARBs (%) | 345 (44.7) |
| Treatment regimen (%) |  |
| One pill, single drug | 368 (47.7) |
| One pill, combination | 186 (24.1) |
| Several pills | 210 (28.2) |

Results are expressed as mean ± standard deviation or as number of participants and (percentage). § defined as the practice of leisure time physical activity at least twice per week. BMI, body mass index; SBP, systolic blood pressure; DBP, diastolic blood pressure; CCB, calcium channel blockers; ACE, angiotensin converting enzyme; ARB, angiotensin receptor blockers.

**Supplementary table 2**: Comparison of the proportion of continuers, combiners, switchers, and discontinuers in several studies.

| **Author** | **Country** | **Publication year** | **Study duration (months)** | **Sample size** | **Continuers** | **Combiners** | **Switchers** | **Discontinuers** |
| --- | --- | --- | --- | --- | --- | --- | --- | --- |
| Degli-Eposti | Italy | 2002 | 12 | 16,783 | 21.8% | 5.1% | 8.2% | 64.9% |
| Mazzaglia [7] | Italy | 2005 | NA | 13,303 | 19.8% | 22.1% | 15.4% | 42.6% |
| Current study | Switzerland | 2012 | 66 | 772 | 54.4% | 26.9% | 12.7% | 6.0% |

NA, not available

**Supplementary table 3:** Persistence with initial treatment in different studies

| **Author** | **Country** | **Publication year** | **Study duration (months)** | **Sample size** | **ARBs** | **ACEIs** | **CCBs** | **BBs** | **Diuretics** |
| --- | --- | --- | --- | --- | --- | --- | --- | --- | --- |
| Bourgault [6] | Canada | 2005 | 36 | 21,326 | 53.0% | 40.0% | 38.0% | 34.0% | 29.0% |
| Degli-Eposti | Italy | 2002 | 12 | 16,783 | 41.7% | 32.2% | 26.7% | 36.9% | 25.9% |
| Erkens [23] | The Netherlands | 2005 | 12 | 17,113 | 62.0% | 59.7% | 34.7% | 35.0% | 33.0% |
| Lachaine [15] | Canada | 2008 | 24 | 4,561 | 60.9% | 58.9% | 64.3% | 69.3% | 52.8% |
| Veronesi [16] | Italy | 2007 | 24 | 347 | 68.5% | 64.5% | 51.6% | 44.8% | 34.4% |
| Current study | Switzerland | 2012 | 66 | 772 | 93.8% | 76.5% | 71.4% | 70.3% | 66.7% |

ACEIs, angiotensin converting enzyme inhibitors; ARBs, angiotensin receptor blockers; BBs, beta-blockers; CCBs, calcium channel blockers.
